# A Comprehensive Evaluation of Soybean Germplasm Resources for Salt Tolerance During Germination

**DOI:** 10.3390/plants14050791

**Published:** 2025-03-04

**Authors:** Lei Han, Lerong Ge, Lin Fei, Chengwei Huang, Yilin Li, Wentan Fan, Dan Zhu, Longgang Zhao

**Affiliations:** 1 College of Grassland Science, Qingdao Agricultural University, Qingdao 266109, China; 2 College of Life Sciences, Qingdao Agricultural University, Qingdao 266109, China; 3 Bathurst College, Qingdao Agricultural University, Qingdao 266109, China; 4 High-Efficiency Agricultural Technology Industry Research Institute of Saline and Alkaline Land of Dongying, Qingdao Agricultural University, Dongying 257300, China; 5 Yunnan Key Laboratory of Crop Wild Relatives Omics, Kunming 650201, China

**Keywords:** salt stress, soybean, germination, screening method, half-lethal concentration

## Abstract

Salt stress impedes normal development, compromises plant quality, and reduces crop yield. The germination phase in soybean marks the initial stage of its growth cycle. Characterizing salt tolerance during this period can help stimulate soybean growth in natural environments and aid the rapid screening of salt-tolerant soybean varieties. Our study characterized the salt tolerance of 36 soybean germplasms in culture dishes during the germination period. Soybeans were subjected to varying concentrations (0, 60, 120, and 180 mmol/L) of NaCl solution to simulate diverse levels of salt stress, and parameters such as germination energy, germination rate, and root length were measured. Statistical techniques such as analysis of variance, membership function, cluster analysis, and quadratic regression equations were used, and the salt tolerance of these 36 soybean germplasms was determined. The critical indicators and the most effective screening concentration for assessing the germination salt tolerance of soybean were identified. Soybeans tolerated low salt concentrations; however, salt concentrations greater than 120 mmol/L significantly inhibited germination indicators. The germination rate, germination vigor, vitality index, seed germination index, total fresh weight, and total dry weight could be used to identify salt tolerance. The semi-lethal concentration of soybean was 155.4 mmol/L, and the coefficient of variation was 20.00%, indicating that it could be used as a screening concentration for evaluating salt tolerance during soybean germination. A total of 36 soybean varieties were classified into four salt tolerance levels through cluster analysis. QN-27, QN-35, and QN-36 were highly salt-resistant materials, and QN-2, QN-17, and QN-19 were salt-sensitive materials. Characterizing salt tolerance during soybean germination can facilitate the selection and breeding of salt-tolerant soybean varieties. Future research utilizing this approach can aid in the selection of soybean varieties with salinity tolerance.

## 1. Introduction

Soybean (*Glycine max*) is an annual dicotyledonous crop in the legume family. It is native to China and was domesticated from the wild soybean (*Glycine soja*) around 6000–9000 years ago [1,2]. Soybean is a globally important crop, providing more than half of the world’s vegetable oil and nearly a quarter of the world’s plant protein. This dual contribution is key for ensuring national food security and enriching the human diet. Due to China’s swift economic expansion and improved living standards, the appetite for meat, eggs, and milk has fueled an exponential increase in the demand for soybeans [3,4]. China imported 99.41 million tons of soybeans, indicating an 11.4% increase from 2022 to 2023. Local soybean production accounts for less than 20% of the total demand, which highlights a substantial disparity between production and consumption [5]. Mitigating soybean imports and lessening external soybean reliance stand as the primary challenge confronting China. Consequently, enhancing soybean yield, expanding cultivation areas, and enhancing overall production are key to promoting China’s soybean industry.

Salt stress is a significant form of abiotic stress, with major economic implications for agricultural production [6,7]; moreover, it is estimated to negatively affect, impacting over 800 million hectares of land globally, including 32 million hectares of agricultural land [8,9]. Recent projections suggest that soil salinization will contribute to 50% of land degradation by 2050 [10]. Salt stress poses a major threat to plant growth and productivity, affecting all developmental stages ranging from seed germination and seedling emergence to flowering and fruit set. It induces a reduction in both crop yield and quality. Salt stress in plants often lends to root dehydration, suppressed ion absorption, decreased photosynthetic efficacy in leaves, and stunted growth, which significantly affects yield and quality formation [11,12]. The development of salt-tolerant crops that can withstand high salinity is one of the most effective biological strategies for coping with this problem and ensuring sustained food production [13,14]. Plants can either be classified as salt-sensitive or salt-tolerant based on their capacity to withstand varying salt concentrations. High salt levels can induce toxicity since specific elements such as sodium cannot be readily regulated within cells. Furthermore, interactions between salt and nutrients may result in nutritional deficiencies [15]. In contrast to abiotic stresses, such as drought and low temperatures, salt stress is prevalent during the reproductive phase of soybean, and the salt tolerance of soybean varies across different growth stages including germination, seedling emergence, flowering, and maturation. There is no apparent correlation between the levels of salt tolerance observed at distinct reproductive stages [16,17]. Previous research indicates that plants are most vulnerable to salt stress during the germination and seedling stages. In practical agricultural settings, ensuring robust and healthy crop seedlings is essential for achieving high and consistent yields. Thus, the ability of soybean seeds to germinate successfully under salt stress is essential for ensuring subsequent growth and development [18,19]. The effect of salinity on seed germination can stem from ionic toxicity or osmosis triggered by a decline in the water potential in the seed’s vicinity. This increase in the osmotic pressure of the soil solution hampers the uptake of water, which impedes germination initiation. In addition, the accumulation of salts in the cells leads to the inactivation of enzymes involved in the seed germination process and hinders the breaking of seed dormancy [20,21].

The Huang–Huai–Hai region is the primary soybean production area in China, and soil salinization, which is primarily driven by sulfates and chlorides, is a major challenge in this region [22]. The salt composition of salinized soils in the Huang–Huai–Hai region has been analyzed in previous studies, and various concentrations of NaCl solutions have been used to replicate the salt stress environment in areas to determine the salt tolerance of diverse soybean germplasm resources. A study conducted by Gobade et al. utilized K-means clustering to analyze the germination indexes of 198 soybean germplasm genotypes, and substantial variation in salt tolerance across these genotypes was revealed. The salt-tolerant genotypes could germinate at concentrations as high as 200 mM. In contrast, the salt-sensitive genotypes failed to germinate, even at a concentration of 100 mM [7]. Cao et al. [23] assessed the salt tolerance of 51 Indonesian soybean germplasms through hydroponic methods and conducted a detailed analysis of six varieties with strong salt tolerance. The findings revealed a notable positive correlation between the expression levels of the salt tolerance gene *Ncl* and the salt tolerance levels observed in soybean varieties. Zhou et al. [24] assessed the salt tolerance levels of 20 distinct soybean varieties throughout the germination phase and determined that the optimal screening concentration of NaCl was 164.50 mmol/L^−1^. An analysis with the affiliation function and cluster analysis techniques revealed that Dongnong 254, Heike 123, Heike 58, Heihe 49, and Heike 68 were salt-tolerant varieties, and Xihai 2, Suinong 94, Kenfeng 16, and Heinong 84 were salt-sensitive varieties. Yu et al. [25] devised a robust and validated methodology for assessing the salt tolerance of alfalfa. This methodology involved the evaluation of 14 traits during the seedling stage of 20 distinct alfalfa varieties. This methodology involves the use of principal components, affiliation functions, clustering techniques, and stepwise regression analysis. Li et al. [26] assessed the salt tolerance of 552 sunflower germplasms with diverse genetic backgrounds. Of these, 30 were classified as highly salt-tolerant germplasms, and 23 were categorized as highly salt-sensitive materials.

Soybean exhibits moderate salt tolerance and a soil salinity threshold of 5.0 ds/m. The cultivation of germplasm acclimated to adverse conditions is a major method for determining the productivity potential of saline soils [25]. Characterizing the impact of salt stress on soybeans has significant theoretical and practical value for the cultivation of salt-tolerant soybean germplasm, enhancing soybean yield and quality, optimizing land use, boosting agricultural economic returns, and fostering the sustainable growth of agriculture. Hence, developing a comprehensive approach for assessing salt tolerance during soybean germplasm germination is crucial for obtaining high-performing salt-tolerant germplasm resources. In this study, 36 soybean germplasm materials were subjected to stress treatments with different concentrations of NaCl (0 mmol/L, 60 mmol/L, 120 mmol/L, and 180 mmol/L), and nine related phenotypic traits, such as germination potential, germination rate, and hypocotyl length, were evaluated to establish a highly efficient and reliable method for characterizing salt tolerance during germination in soybeans. This approach permits salt-tolerant soybean germplasm resources to be identified and provides a reference for studies of salt tolerance and the selection of new germplasm.

## 2. Results

### 2.1. The Effects of Different Concentrations of NaCl on Various Indicators of Soybean Germination Stage

**Figure 1:**
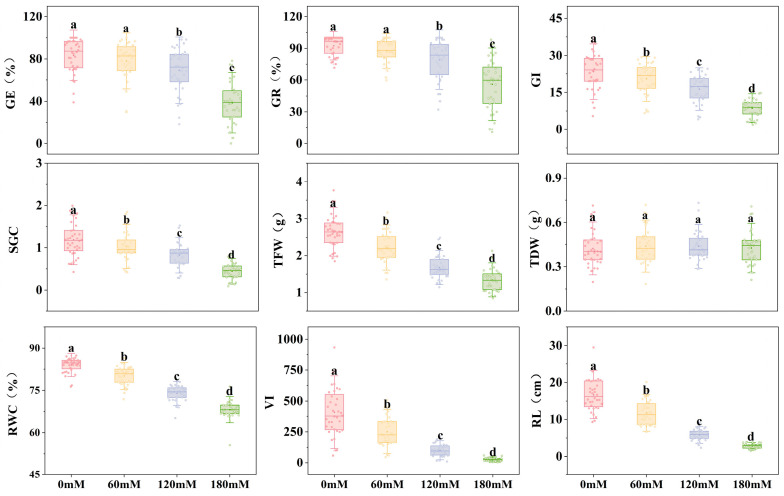
Comparison of indicators of soybean germination under different levels of salt stress. Note: The germination performance of 36 soybean germplasms under varying levels of salt stress, GR: germination rate; GE: germination energy; SGC: seed germination coefficient; GI: germination index; TFW: total fresh weight; TDW: total dry weight; RL: root length; RWC: relative water content; VI: vigor index. The different letters in the figure indicate significant differences (*p* < 0.05).

Salt tolerance assessment was performed during the germination of 36 soybean germplasms using NaCl solutions of varying concentrations (Figure 1 and Table 1). The germination energy and germination rate of soybeans remained relatively stable at 60 mM compared with the control group, indicating that soybean seeds exhibited moderate tolerance to lower levels of salt stress. However, the germination index, seed germination coefficient, total fresh weight, relative water content, vigor index, and root length consistently declined as salt concentrations increased. At concentrations greater than 120 mM NaCl, this decline became more pronounced, indicating that salt stress substantially inhibited germination indicators, and its effect was more pronounced at higher concentrations. The vigor index and radicle length were the most affected by increasing salt levels. The vigor index was 38.48%, 75.32%, and 93.79% lower, and the radicle length was 29.48%, 65.07%, and 83.28% lower at 60 mM, 120 mM, and 180 mM, respectively, compared with the control. This highlights the sensitivity of the vigor index and root length as indicators of the effect of salt stress on soybean germination.

The coefficient of variation for each indicator of the test materials differed at varying salt concentrations, indicating that the sensitivity to salt stress associated with these indicators was inconsistent. With the exception of embryonic root length, the coefficient of variation for each index was highest at 180 mM, followed by 120 mM, 60 mM, and 0 mM. This trend indicates that salt stress magnifies the disparities among soybean varieties, which facilitates the selection of germplasm with enhanced salt tolerance.

### 2.2. Multifactor Analysis of Variance of Each Indicator

Multifactor analysis of variance (ANOVA) was used to examine the independent and interactive effects of two factors on the measured indexes: soybean germplasm and NaCl concentrations (Table 2). The results indicated that, aside from the total dry weight, the F-values corresponding to various NaCl concentrations were significantly greater than 0.5, and the F-values for each index across different germplasms and NaCl concentrations were all below 0.01. This outcome highlights the substantial effect of different soybean varieties and NaCl concentrations on the measured indexes. Furthermore, a notable reciprocal influence between the varieties and NaCl concentrations was observed, and the significance levels of all corresponding F-values were below 0.01.

### 2.3. Principal Component Analysis of the Salt Tolerance Coefficient

The outcomes of the principal component analysis for the salt tolerance coefficients of the nine indexes are shown (Table 3). Three principal components, each possessing eigenvalues exceeding 1, were obtained. Principal components with eigenvalues greater than 1 corresponded to components with a variance larger than that of the original variables; these principal components could thus better explain the variance in the original data and better capture changes and patterns in the data. Their cumulative contributions amounted to 80.157%, 84.746%, and 82.928% at NaCl concentrations of 60 mM, 120 mM, and 180 mM, respectively. This helped elucidate variation observed in the nine traits under consideration.

At a concentration of 60 mM NaCl, the eigenvalue of CS1 was 3.484. This value was mainly correlated with three indicators, RRWC, RVI, and RRL, which explained 38.487% of the variation in the data. This suggests that the relative water content, vigor index, and radicle length serve as crucial indicators for assessing salt tolerance at 60 mM NaCl. With an eigenvalue of 2.114, CS2 was mainly correlated with RGE, RGR, RGI, and RSGC and explained 23.493% of the variation in the data. This component chiefly delineates the overall germination of soybeans under 60 mM NaCl stress. CS3 exhibited an eigenvalue of 1.616 and was primarily correlated with RTFW and RTDW; it explained 17.956% of the variation in the data. This component mainly reflects alterations in soybean biomass. At a concentration of 120 mM NaCl, the eigenvalue of CS1 was 3.395. CS1 was mainly correlated with four indicators, RGE, RGR, RGI, and RSGC, which explained 37.728% of the variation in the data. This component chiefly delineates the overall germination process of soybeans under 120 mM NaCl stress, which makes it a crucial indicator for evaluating salt tolerance at this specific concentration. With an eigenvalue of 2.623, CS2 was mainly correlated with three indicators, RRWC, RVI, and RRL, which explained 29.141% of the variation in the data. This component was mainly associated with changes in the embryonic root length of soybeans under stress. CS3 had an eigenvalue of 1.609, and it was primarily composed of two indicators, RTFW and RTDW, which explained 17.877% of the variation in the data. This component was mainly associated with changes in soybean biomass. At a concentration of 180 mM NaCl, the eigenvalue of CS1 was 3.727. This component was primarily correlated with three indicators, RGE, RGR, and RSGC, and explained 41.414% of the variation in the data. This component was mainly associated with the overall sprouting process of soybeans under 180 mM NaCl stress; it thus serves as a crucial indicator for evaluating salt tolerance at this particular concentration. With an eigenvalue of 2.020, CS2 was mainly correlated with two indicators, RVI and RRL, which explained 22.449% of the variation in the data. This component mainly responded to changes in the embryonic root length of soybeans under stress. CS3 had an eigenvalue of 1.716 and was mainly correlated with two indicators, RTFW and RTDW, which explained 19.065% of the variation in the data. This component mainly responded to changes in soybean biomass.

The principal component analysis of salt tolerance coefficients at varying concentrations highlighted that RWT, VI, and RL are pivotal indicators for evaluating and identifying salt tolerance in soybeans under mild (<60 mM NaCl) salt stress. However, overall changes in integrated sprouting and biomass were insufficient for fully capturing the effect of salt stress on soybean germination. As salt concentrations increased, integrated sprouting increasingly influenced soybean germination, and GE, GR, and SGC were the key indexes for characterizing salt tolerance during soybean germination under moderate and severe salt stress.

### 2.4. Analysis of the Affiliation Function

The weight coefficients were 40.52%, 33.4%, and 26.08% for the three composite indexes in the 60 mM NaCl treatment. Similarly, in the 120 mM NaCl treatment, the weight coefficients were 44.518%, 34.386%, and 21.095% for the three composite indexes. At 180 mM NaCl, the weight coefficients were 49.94%, 27.07%, and 22.99%, which indicates that each principal component was significant at each salt concentration (Table 3). Consequently, by integrating the affiliation function values of each composite index across different germplasms with the respective weight coefficients, the composite evaluation values (D-values) representing the salt tolerance of diverse soybean germplasm under distinct NaCl concentrations were calculated.

The degree of affiliation (μn) and composite evaluation value (D-value) for each trait in 36 soybean germplasm were calculated based on the three principal component factor scores derived from the principal component analysis (Tables S1–S3). The maximum D-value recorded was 0.93 (QN-27), and a minimum of 0.21 (QN-17) was observed in the 60 mM NaCl treatment. Additionally, the maximum D-value reached 0.83 (QN-15) with a minimum of 0.35 (QN-17) in the 120 mM NaCl treatment. In the 180 mM NaCl treatment, the D-value peaked at 0.90 (QN-16) and had a minimum of 0.23 (QN-19).

The varied ranking of D-values under distinct NaCl treatments stems from the differential effects of varying concentrations on soybeans, which reflects diversity in the adaptive traits across various soybean germplasms. Moreover, given the differing weights of composite indexes under various salt concentrations, the indices should be analyzed across varying salt concentrations.

### 2.5. Correlation Analysis

**Figure 2:**
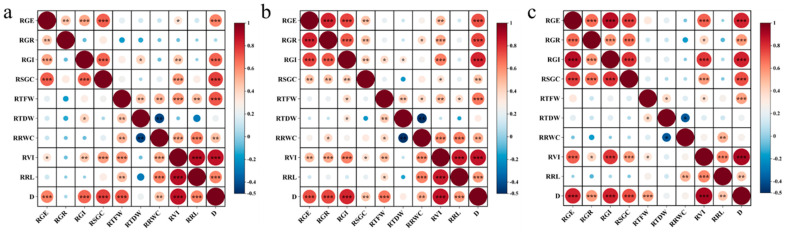
Correlation analysis between various indicators and D-values during soybean germination under different degrees of salt stress. Note: The Pearson correlation coefficient method was employed to perform a correlation analysis on indicators from 36 soybean germplasm accessions: * indicates a significant correlation at the 0.05 level, ** indicates a significant correlation at the 0.01 level, and *** indicates a significant correlation at the 0.001 level. (**a**) The correlation between salt tolerance coefficient and D-value during soybean germination under 60 mmol/L NaCl stress; (**b**) the correlation between salt tolerance coefficient and D-value during soybean germination under 120 mmol/L NaCl stress; (**c**) the correlation between salt tolerance coefficient and D-value during soybean germination under 180 mmol/L NaCl stress.

An analysis of correlations between the relative salt tolerance index and the D-value of soybean germination under varying NaCl concentrations was conducted (Figure 2). In Figure 2a, there was no significant correlation between RGR and the D-value, and the significance of the correlation between RGR and the D-value gradually increased as the salt concentration increased, indicating that the effect of low salt stress on soybean germination was small; however, the strength of this effect increased with the salt concentration. Figure 2c revealed no significant correlation between the RRWC and D-value, indicating that RRWC cannot be used as an index to identify the salt tolerance of soybean under heavy salt stress. Analysis of Figure 2a–c revealed no significant correlation between RTDW and the D-value under different concentrations of salt stress, implying that TDW is not a key indicator of soybean salt tolerance. In addition, RGE, RGR, RGI, and RSGC were correlated with RTFW, RTDW, and RR, indicating that soybean germination and biomass were independent and that the phenotypes were all affected under salt stress.

### 2.6. Regression Analysis

Initially, the 0, 60, 120, and 180 mM NaCl concentrations were simplified to 0, 1, 2, and 3, respectively. Selected indicators at different salt levels were analyzed through quadratic regression using the relative values of each indicator at varying salt concentrations to derive equations and their coefficients (Table S4). Varieties with negative coefficients, as determined by the characteristics of the one-variable quadratic equation, exhibited a growth-promoting trend under low NaCl stress. Specifically, negative coefficients for the RGR, RGE, and RGI indexes were observed for over half of the varieties, indicating that low NaCl concentrations had the most pronounced positive effect on seed germination.

By utilizing the formula y = ax^2^ + bx + c along with the coefficients provided in Table S4, the most precise screening concentrations for each germplasm were determined by predicting the half-lethal concentration (LC_50_) of each indicator at y = 0.5. The results are shown in Table 4. The mean LC_50_ value for different soybean germplasms was 2.59, which is equivalent to 155.4 mmol/L, and the coefficient of variation was 0.20. LC_50_ values under RVI and RRL were relatively low at 1.30 and 1.59, which corresponded to 78.00 mmol/L and 95.40 mmol/L, with coefficients of variation of 0.28 and 0.25, respectively. This suggests that the vigor index and radicle length were more sensitive to salt stress. LC_50_ values of RGE, RGR, and RTFW were relatively high, with mean values of 3.02, 3.90, and 3.01; corresponding values of 181.20 mmol/L, 234.00 mmol/L, and 180.60 mmol/L; and coefficients of variation of 0.24, 0.48, and 0.17, respectively. Significant variation was observed in the LC_50_ values among different germplasms and indicators. Consequently, the ideal screening concentration of NaCl for soybean germplasm was determined through the averaging of all LC_50_ values across germplasms.

### 2.7. Cluster Analysis

**Figure 3:**
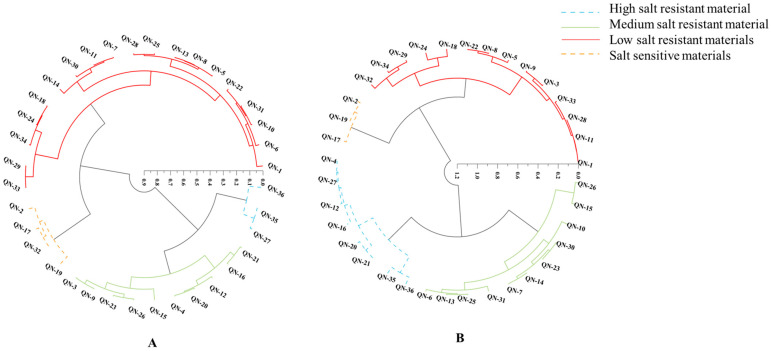
Cluster analysis of the salt tolerance of 36 soybean germplasms under NaCl stress. Note: (**A**) cluster analysis under all salt concentrations; (**B**) LC_50_ cluster analysis. Hierarchical cluster analysis was performed using the D-value and Origin; the coordinate axis represents the Euclidean distance.

A comprehensive assessment of plant salt tolerance during germination requires consideration of both seed germination under salt stress and subsequent seedling growth. Data from a single NaCl concentration may not accurately and realistically portray the salt tolerance of soybean germplasm, given the substantial variability in salt tolerance among varieties exposed to varying NaCl concentrations. The salt tolerance of 36 soybean germplasms was assessed through clustering based on mean values of the affiliation function values of RGR, RGE, RGI, RSGC, RTFW, RVI, and RRL across different salt stress levels. Using an Euclidean distance of 0.3, the 36 soybean germplasms were categorized into four distinct classes (Figure 3A). The figure showed that QN-27, QN-35, and QN-36 were highly salt-resistant materials; 10 germplasms, including QN-3, QN-4, and QN-9, were medium salt-resistant materials; 13 germplasms, such as QN-1, QN-6, and QN-33, were low salt-resistant materials; and QN-2, QN-17, QN-19, and QN-32 were salt-sensitive materials.

The analyzed LC_50_ of 2.59 was introduced into the regression equation to obtain the relative values of each index under LC_50_. The relative values of each index were used to perform an affiliation function analysis, and the obtained affiliation function values (Table S5) were used for cluster analysis to obtain Figure 3B. At an Euclidean distance of 0.6, the 36 soybean germplasms were classified into four categories. Eight materials, such as QN-35, QN-36, and QN-27, were highly salt-resistant materials; 11 germplasms, such as QN-6, QN-7, and QN-10, were medium salt-resistant materials; QN-1, QN-11, QN-18, and 14 other materials were low salt-resistant materials; and QN-2, QN-17, and QN-19 were salt-sensitive materials. The use of both clustering methods indicated that QN-27, QN-35, and QN-36 were highly salt-resistant materials, and QN-2, QN-17, and QN-19 were salt-sensitive materials.

## 3. Discussion

### 3.1. The Effect of Different Concentrations of NaCl on the Soybean Germination Period

Salt stress significantly inhibits plant growth, with the extent of growth reduction influenced by multiple factors, including plant species, developmental stage, and salt concentration [27,28]. Over the past three decades, many regulatory substances contributing to salt tolerance involved in stress sensing, signal transduction, ion transport, metabolic adaptation, and growth responses have been identified [29]. The presence of Na^+^ and Cl^−^ ions induces lipid peroxidation in the cell membrane of the seed coat, which induces structural damage to plant cell membranes and interferes with cell division and other crucial activities. This process diminishes the permeability of the seed coat, which impedes the penetration of water into the cotyledon and hypocotyl, ultimately lowering the seed germination rate [30,31,32]. Salt stress disrupts the ionic balance in plants, and the excessive penetration of Na^+^ into plant cells hampers the uptake of essential ions such as K^+^, Ca^2+^, and Mg^2+^, which has detrimental effects on the cell membrane system, ion channels, and regulatory processes, thereby impairing plant physiological and metabolic functions [33,34,35]. Our findings indicated that there were no notable alterations in the germination potential and rate in the presence of low salt concentrations. However, the salt tolerance coefficient of specific salt-tolerant materials was considerably increased, suggesting that low salt concentrations facilitate seed water absorption and metabolism, thereby enhancing seed germination. The decline in all indicators became more pronounced when the NaCl concentration exceeded 120 mM. The germination index, seed germination index, total fresh weight, relative water content, vitality index, and embryonic root length substantially decreased as the salt concentration increased. Variance analysis was performed on the salt tolerance coefficients of diverse indicators at varying concentrations, which revealed significant interaction effects between distinct germplasm types and NaCl concentrations. Some inconsistencies were observed in the results of the principal component analysis for soybean germplasm under different NaCl concentrations, including variation in the ranking of membership functions across lineages; these findings highlight the diversity in salt tolerance among soybean germplasms.

### 3.2. Determination of Key Indicators and Screening Concentration for Salt Tolerance Identification and Determination

Researchers commonly utilize germination energy and germination percentage for assessing salt tolerance during soybean germination [36]. Wang et al. [37] used a concentration gradient spanning 0, 30, 60, 90, 120, 150, and 180 mM NaCl to evaluate the optimal stress concentrations for salt tolerance in soybean germination. Through one-way ANOVA, principal component analysis, and correlation analysis, significant variability was observed in root length and the vitality index under low salt stress. As the salt concentration increased, the correlation between soybean germination-related indicators and the D-value increased, which emphasizes that soybean germination indicators are key for assessing soybean salt tolerance under high salt stress. Based on the aforementioned analysis, germination energy, germination rate, seed germination coefficient, root length, germination index, total fresh weight, and vigor index can be used to characterize salt tolerance. The semi-lethal concentration (LC_50_) is commonly used to evaluate stress-inducing concentrations of NaCl in plants [38,39]. The average semi-lethal salt concentration in this study was 2.59, indicating that the theoretical optimal NaCl concentration for identifying soybean salt tolerance is 155.4 mmol/L. Shi et al. [40] studied the effect of 150 mmol/L NaCl on soybean germination. Moreover, Ravelombola et al. [41] showed that 150 mmol/L NaCl was a suitable concentration for assessing salt tolerance in cowpea.

### 3.3. Classification of Salt Tolerance in Soybean Germplasm

The D-value in the affiliation function is a composite of all the salt tolerance traits. Hussain et al. screened 40 local wheat varieties under 150 mM NaCl stress [42]. Cluster analysis was performed on the average membership function values of various indicators at different NaCl concentrations. Comprehensive comparisons were made through affiliation function and cluster analysis; QN-27, QN-35, and QN-36 were highly salt-tolerant materials, and QN-2, QN-17, and QN-19 were salt-sensitive materials. Zhou et al. [24] achieved consistent results in screening salt-sensitive and salt-tolerant lines by comparing the affiliation function values of each index under semi-lethal salt stress conditions with the average affiliation function values of the same indices across varying NaCl concentrations. Simultaneously comparing the outcomes of both clusters and conducting a thorough assessment of salt tolerance revealed differences between highly salt-resistant materials and salt-sensitive materials, which are possibly attributed to differences in the resistance of soybean germplasm to salt stress. Some germplasms exhibit strong salt tolerance under low salt stress, while others show strong salt tolerance under high salt stress. Relying on the average values of various indicators under different salt stress levels to screen salt-tolerant germplasms may fail to account for salt tolerance information obtained before and after salt stress.

The method of salt tolerance identification in soybean used in this study can provide methodological support for subsequent researchers, ideas that could aid assessments of salt tolerance in soybean or other crops, and information for genetic research or marker-assisted selection strategies for enhancing salt tolerance. We also combined the experimental results obtained from assessments of salt tolerance during the germination stage of soybean with field planting in saline swamps and other environments, which helps validate the results of field studies. Our approach to assessing salt tolerance can facilitate field-based studies aimed at characterizing the adaptability and growth performance of salt-tolerant varieties in areas with saline soil; this has implications for the breeding of salt-tolerant plants.

## 4. Materials and Methods

### 4.1. Materials

The 36 soybean lines utilized for testing were sourced from the Laboratory of Molecular Genetics and Crop Phenotypic Breeding of Soybean at Qingdao Agricultural University. Detailed information regarding these varieties (lines) can be found in the attached Table S6.

### 4.2. Experimental Design

The experiment took place in October 2023 at the Laboratory of Molecular Genetics and Crop Phenotype Breeding of Soybean at Qingdao Agricultural University. Distilled water served as the control (0 mM), while solutions of 60 mmol/L NaCl simulated mild salt stress (60 mM), 120 mmol/L NaCl represented moderate salt stress (120 mM), and 180 mmol/L NaCl induced severe salt stress conditions (180 mM).

High-quality soybean seeds, free from insect infestation and physical damage, were meticulously selected and placed in Petri dishes within a desiccator. A sterilization process was initiated by adding a 5.25% sodium hypochlorite solution along with 5 mL of concentrated hydrochloric acid (36%) to generate chlorine gas gradually. This setup was maintained in a fume cupboard for 16 h to ensure effective sterilization. Post-sterilization, the seeds were rinsed 2–3 times with distilled water to remove any residual chemicals. They were then arranged in square Petri dishes, each lined with a double layer of filter paper. For consistency, twenty-five seeds were evenly distributed in each dish, and this was repeated three times. The Petri dishes were incubated at a constant temperature of 25 °C in an incubator for 7 days. Observations were made every 24 h to monitor germination progress, with the criterion for successful germination being root growth reaching at least half the length of the seed. To maintain optimal moisture levels, the culture solution was replenished daily with a solution of equal concentration. During the germination test, any seeds showing signs of mold were promptly removed and recorded to prevent contamination. Additionally, if any culture solution developed an unusual odor, it was immediately replaced with fresh solution to ensure the integrity of the germination environment. In cases where mold growth was evident on the germination bed, the affected bed was replaced immediately to maintain a sterile and conducive environment for seed germination.

This meticulous approach ensured accurate monitoring and documentation of the germination process over the 7-day period, providing reliable data on the germination rate and health of the soybean seeds.

The amount of germination was investigated every day until the seventh day. The related phenotypic indexes and formulas were determined as follows (see Supplementary Table S7 for the meaning of the acronyms):

Germination Energy (GE), GE/% = number of germinated seeds on day 4/number of seeds for test × 100;

Germination Rate (GR), GR/% = number of germinated seeds on day 7/number of seeds for test × 100;

Germination Index (GI), GI = ∑(Gt/Dt), (Gt is the number of germinated seeds on day t, Dt is the corresponding number of days of germination);

Seed Germination Coefficient (SGC), SGC = 1.00 × nd2 + 0.70 × nd4 + 0.30 × nd6, (nd2, nd4, nd6 refer to the germination rate of seeds on days 2, 4, and 6, respectively);

Three representative bean sprouts were selected on day 7 to measure their total fresh weight (TFW) and root length (RL); the samples were baked in an oven at 80 °C until constant weight, and their total dry weight (TDW) was determined:

Relative Water Content (RWC), RWC/% = (TFW − TDW)/TFW × 100;

Vigor Index (VI), VI = GI × RL;

Salt tolerance Coefficient (SC) = treatment value/control value.

### 4.3. Data Statistics and Analysis

The data were organized using Microsoft Excel 2021. Origin 2021 software was utilized for data plotting and systematic cluster analysis. IBM SPSS Statistics 26.0 was employed for tasks such as principal component analysis, Pearson correlation analysis, analysis of variance, affiliation function analysis, and quadratic regression analysis. Through these methodologies, the salt tolerance of the test materials was comprehensively assessed.

The formula of the affiliation function analysis was as follows:

The degree of affiliation μ(Xi) is calculated as follows: μ(Xi) = (Xi − Xmin)/(Xmax − Xmin), where Xi represents the ith composite index, Xmin denotes the minimum value of the ith composite index, and Xmax signifies the maximum value of the ith composite index.

The weight of each composite indicator (Wi) is determined by the following formula: Wi = Pi/(∑Pi), where Wi represents the weight of the ith composite indicator among all composite indicators, and Pi stands for the contribution rate of the ith composite indicator for each variety or line.

The comprehensive evaluation value (D-value) is computed as: D-value = ∑[μ(Xi) × Wi], where D-value signifies the comprehensive evaluation value of salt tolerance of varieties or lines calculated using the comprehensive indexes under salt stress. A higher D-value indicates superior composite traits.

Following the calculation of the affiliation function values for each soybean germplasm under various salt concentrations, the average value was derived, and systematic cluster analysis was conducted based on this average value. Subsequently, a one-way quadratic regression analysis of the relative values of each index concerning salt concentration was conducted using SPSS 26.0, represented by the equation y = ax^2^ + bx + c, where a, b, and c denote the equation coefficients.

Utilizing the obtained quadratic regression equation, the LC_50_ concentration (the salt concentration under semi-lethal conditions) for each relative index at Y = 0.5 was determined to assess the salt tolerance level of different soybean germplasms under semi-lethal conditions. The arithmetic mean of the LC_50_ values was calculated to ascertain the LC_50_ concentration, and these mean LC_50_ values were incorporated into a quadratic equation to derive the relative values of each index under the LC_50_ concentration. Subsequently, an affiliation function analysis was performed on these values, followed by a systematic cluster analysis. The results of both cluster analyses were amalgamated to validate the identified concentrations for a more comprehensive evaluation of the salt tolerance levels among different varieties.

## 5. Conclusions

In this study, we evaluated the salt tolerance of different soybean germplasms at the germination stage and identified soybean materials suitable for growth in saline soil. Soybeans showed resistance to low salt stress in the germination stage, and the strength of the inhibitory effect on soybean seed germination increased as the NaCl concentration increased. One-way ANOVA, principal component analysis, and correlation analysis indicated that GR, GE, GI, RVI, SGC, TFW, and RL can be used to characterize salt tolerance in the germination stage of soybeans. Based on the regression analysis, the semi-lethal salt concentration for soybean was 155.4 mmol/L. Comprehensive comparisons were made through affiliation function and cluster analysis; QN-27, QN-35, and QN-36 were highly salt-tolerant materials, and QN-2, QN-17, and QN-19 were salt-sensitive materials. The establishment of a method for evaluating the salt tolerance of soybean during the germination stage can facilitate the selection and breeding of salt-tolerant soybean varieties. In future research, this approach could be used to accelerate the improvement and selection of salt-tolerant soybean varieties. Overall, our findings have major implications for the future breeding and cultivation of salt-tolerant soybean plants.

## Figures and Tables

**Table 1:** Descriptive analysis of indicators of soybean germination under different levels of salt stress.

Treatment	Index	GE/%	GR/%	GI	SGC	TFW/g	TDW/g	RWC/%	VI	RL/cm
0 mM	Max	100.00	100.00	37.13	2.00	3.77	0.71	89.34	934.42	29.49
	Min	39.06	71.67	5.39	0.42	1.85	0.20	76.32	57.00	9.28
	Mean	83.36	92.58	23.45	1.19	2.64	0.42	83.99	408.26	16.69
	SD	15.97	8.96	7.53	0.39	0.44	0.12	2.73	196.27	4.33
	CV	0.19	0.10	0.30	0.33	0.17	0.23	0.03	0.47	0.26
60 mM	Max	100.00	100.00	29.33	1.85	3.17	0.72	86.35	506.23	20.10
	Min	29.81	59.64	6.66	0.42	1.36	0.18	71.90	44.26	6.64
	Mean	78.09	87.66	20.54	1.03	2.22	0.44	80.08	251.17	11.77
	SD	17.67	11.08	6.07	0.35	0.41	0.12	3.19	118.64	3.28
	CV	0.23	0.13	0.32	0.34	0.18	0.26	0.04	0.48	0.28
120 mM	Max	98.33	100.00	26.26	1.53	2.49	0.73	78.49	212.73	9.07
	Min	18.33	32.09	4.10	0.29	1.15	0.29	65.14	9.66	2.36
	Mean	69.31	79.31	16.33	0.83	1.68	0.44	73.93	100.77	5.83
	SD	20.97	18.73	5.77	0.28	0.31	0.10	2.80	51.44	1.51
	CV	0.30	0.24	0.35	0.34	0.18	0.27	0.04	0.51	0.26
180 mM	Max	78.27	98.13	15.79	0.86	2.13	0.71	76.34	58.41	3.90
	Min	0.00	10.83	1.88	0.09	0.85	0.21	55.58	4.60	1.52
	Mean	38.47	56.07	8.65	0.45	1.34	0.43	68.22	25.35	2.79
	SD	19.06	22.94	3.86	0.20	0.31	0.11	3.12	14.14	0.68
	CV	0.50	0.41	0.45	0.45	0.23	0.28	0.05	0.56	0.24

Note: These experimental data are based on the experimental results of 36 soybean varieties (lines) under different salt treatments. The values are the maximum (Max), minimum (Min), mean (Mean), standard deviation (SD), and coefficient of variation (CV) of all indicators of the tested materials at different concentrations. GR: germination rate; GE: germination energy; SGC: seed germination coefficient; GI: germination index; TFW: total fresh weight; TDW: total dry weight; RL: root length; RWC: relative water content; VI: vigor index.

**Table 2:** Multivariate analysis of variance for soybean salt tolerance indicators.

Index		Germplasm	NaCl Concentration	Germplasm and NaCl Concentration
RGE	F	27.176	300.58	2.199
	Sig	<0.01	<0.01	<0.01
RGR	F	22.849	171.023	2.193
	Sig	<0.01	<0.01	<0.01
RGI	F	39.542	497.529	2.376
	Sig	<0.01	<0.01	<0.01
RSGC	F	29.284	469.048	2.124
	Sig	<0.01	<0.01	<0.01
RTFW	F	23.385	612.680	1.989
	Sig	<0.01	<0.01	<0.01
RTDW	F	34.640	1.077	1.473
	Sig	<0.01	>0.05	<0.01
RRWC	F	7.484	443.229	1.224
	Sig	<0.01	<0.01	<0.01
RVI	F	16.378	786.579	3.591
	Sig	<0.01	<0.01	<0.01
RRL	F	17.471	1388.012	5.410
	Sig	<0.01	<0.01	<0.01

Note: F-tests were used for analysis of variance. The F-value in the results represents a specific value obtained by the F-test formula, and the corresponding *p*-value was obtained according to a numerical table. Specifically, sig. A signal (sig) value < 0.05 indicates an influence on the result; otherwise, there was no effect. RGR: relative germination rate; RGE: relative germination energy; RSGC: relative seed germination coefficient; RGI: relative germination index; RTFW: relative total fresh weight; RTDW: relative total dry weight; RRL: relative root length; RRWC: relative water content; RVI: relative vigor index.

**Table 3:** Factor score and contribution rate of each principal component in the germination period.

Item	Trait	60 mM			120 mM			180 mM		
		CS1	CS2	CS3	CS1	CS2	CS3	CS1	CS2	CS3
Eigenverctor	RGE	0.108	0.883	0.119	0.941	0.069	−0.014	0.919	0.009	0.203
	RGR	−0.184	0.657	−0.464	0.899	0.179	−0.046	0.771	−0.234	0.113
	RGI	0.089	0.599	0.642	0.847	0.162	0.299	0.186	−0.037	−0.177
	RSGC	0.306	0.850	0.168	0.815	0.248	0.218	0.897	−0.006	0.020
	RTFW	0.650	0.021	0.591	0.143	0.550	0.655	0.117	0.296	0.867
	RTDW	−0.277	0.053	0.874	0.151	−0.233	0.935	0.201	−0.352	0.878
	RRWC	0.837	−0.001	−0.211	0.241	0.808	−0.361	0.000	−0.172	0.041
	RVI	0.861	0.329	0.203	0.466	0.796	0.190	−0.053	0.833	−0.232
	RRL	0.915	0.075	−0.112	0.037	0.925	0.015	0.001	0.869	0.196
Eigenvalue		3.484	2.114	1.616	3.395	2.623	1.609	3.727	2.020	1.716
Contribution (%)		38.708	23.493	17.956	37.728	29.141	17.877	41.414	22.449	19.065
Cumulative contribution (%)		38.708	62.201	80.157	37.728	66.869	84.746	41.414	63.863	82.928
Weight coefficient (%)		40.520	33.400	26.080	44.518	34.386	21.095	49.940	27.070	22.990

Note: Using principal component analysis, CS1, CS2, and CS3 are the principal component values corresponding to each trait. RGR: relative germination rate; RGE: relative germination energy; RSGC: relative seed germination coefficient; RGI: relative germination index; RTFW: relative total fresh weight; RTDW: relative total dry weight; RRL: relative root length; RRWC: relative water content; RVI: relative vigor index.

**Table 4:** Salt-tolerant half-lethal concentration of soybean.

Germplasm	Salt-Tolerant Half-Lethal							Average Value
	RGE	RGR	RGI	RSGC	RTFW	RVI	RRL	
QN-1	3.16	3.38	2.51	2.20	3.66	0.93	1.17	2.43
QN-2	0.97	1.78	2.29	1.62	2.49	1.29	1.63	1.73
QN-3	3.25	2.52	2.28	2.48	3.76	1.62	2.10	2.57
QN-4	2.89	3.38	2.59	2.96	4.50	2.14	2.54	3.00
QN-5	2.71	2.87	2.36	2.67	2.47	1.65	2.10	2.40
QN-6	2.90	5.67	2.65	3.08	2.70	1.18	1.40	2.80
QN-7	3.70	5.08	2.80	2.52	2.94	1.27	1.58	2.84
QN-8	2.68	2.93	2.57	2.70	2.70	1.23	1.29	2.30
QN-9	2.84	2.96	2.62	1.18	2.99	1.51	1.76	2.27
QN-10	3.43	6.73	2.34	3.92	4.32	1.19	1.90	3.40
QN-11	2.32	2.49	2.38	2.32	2.54	1.90	2.33	2.33
QN-12	3.51	3.90	3.09	2.95	2.73	1.78	1.81	2.82
QN-13	2.85	4.63	2.59	2.94	2.74	1.11	1.36	2.60
QN-14	3.42	3.50	2.99	2.83	3.00	0.49	1.18	2.49
QN-15	2.87	4.53	2.77	2.97	3.07	1.47	1.77	2.78
QN-16	5.74	9.46	3.80	4.06	3.28	1.33	1.66	4.19
QN-17	1.88	1.74	1.66	1.54	2.69	0.90	1.55	1.71
QN-18	2.52	2.54	2.23	2.38	3.18	1.07	1.49	2.20
QN-19	2.54	2.58	2.29	1.97	2.31	0.93	1.13	1.96
QN-20	3.04	11.24	2.54	3.16	3.03	1.72	2.16	3.84
QN-21	3.29	3.47	3.12	2.85	3.18	1.45	1.51	2.69
QN-22	2.82	3.16	2.43	2.79	2.94	1.19	1.48	2.40
QN-23	2.50	2.91	2.64	2.50	3.05	1.46	1.84	2.41
QN-24	2.71	4.03	2.31	2.24	2.86	0.92	1.26	2.33
QN-25	3.77	3.35	3.15	3.51	2.55	1.01	1.22	2.65
QN-26	2.77	3.86	2.75	2.85	2.99	1.68	1.83	2.67
QN-27	3.59	2.34	3.27	3.53	3.34	1.78	1.43	2.75
QN-28	2.79	3.92	2.70	2.04	2.92	1.01	1.37	2.39
QN-29	2.37	2.72	2.33	2.15	2.60	1.37	1.69	2.17
QN-30	3.47	3.48	3.01	3.35	3.40	0.86	0.93	2.65
QN-31	3.23	3.32	2.70	2.38	2.87	1.06	1.33	2.41
QN-32	2.85	3.59	2.34	2.18	2.47	0.89	1.11	2.20
QN-33	2.82	3.53	2.65	2.46	2.39	1.07	1.22	2.31
QN-34	2.67	2.89	2.46	2.43	2.27	1.06	1.14	2.13
QN-35	4.09	5.06	2.75	2.86	3.90	2.03	2.47	3.31
QN-36	3.85	4.96	3.42	3.08	3.65	1.41	1.65	3.15
Mean	3.02	3.90	2.65	2.66	3.01	1.30	1.59	2.59
CV	0.24	0.48	0.15	0.23	0.17	0.28	0.25	0.20

Note: The values are the half-lethal salt concentrations x when each indicator is 0.5 (Y = 0.5), obtained from the quadratic function (Y = ax^2^ + bx + c) in Supplementary Table S5.

## Data Availability

Data are contained within the article and Supplementary Materials.

## Supplementary Materials

The following supporting information can be downloaded at: https://www.mdpi.com/article/10.3390/plants14050791/s1, Table S1: Membership function values and comprehensive ranking of soybean germination stage under 60 mM NaCl concentration; Table S2: Membership function values and comprehensive ranking of soybean germination stage under 120 mM NaCl concentration; Table S3: Membership function values and comprehensive ranking of soybean germplasm under 180 mM NaCl concentration; Table S4: Coefficient table of quadratic regression equation in one variable; Table S5: Membership function values and comprehensive ranking of soybean germplasm under semi lethal concentration (LC_50_); Table S6: 36 soybean germplasm names;
