# Supplementary material for: A Comprehensive Evaluation of Soybean Germplasm Resources for Salt Tolerance During Germination
Source: Plants (Basel). 2025 Mar 4;14(5):791. doi: 10.3390/plants14050791 (PMC11902203; doi:10.3390/plants14050791)
Supplement: Supplementary file 1 [file plants-14-00791-s001.zip › plants-3436211-supplementary.pdf]

Table S1. Membership function values and comprehensive ranking of soybean germination stage under 60mM NaCl concentration

Germplasm name	Comprehensive index			Membership function			D-value	Sort
	F1	F2	F3	u1	u2	u3		
QN-1	-0.80	-0.48	-0.12	0.27	0.35	0.44	0.34	30
QN-2	0.70	-3.02	1.03	0.65	-0.28	0.73	0.36	29
QN-3	1.02	0.84	-0.46	0.73	0.68	0.36	0.61	6
QN-4	1.97	0.11	-1.40	0.96	0.50	0.13	0.59	8
QN-5	0.81	1.33	-0.87	0.67	0.80	0.26	0.61	7
QN-6	0.02	0.15	-0.49	0.48	0.51	0.35	0.46	19
QN-7	-0.03	-0.01	-0.24	0.47	0.47	0.41	0.45	22
QN-8	-0.95	2.17	-0.52	0.24	1.01	0.35	0.52	10
QN-9	0.32	-0.40	-0.57	0.55	0.37	0.33	0.43	24
QN-10	0.18	-0.79	-2.38	0.52	0.28	-0.12	0.27	35
QN-11	2.13	-0.64	0.91	1.00	0.31	0.70	0.69	2
QN-12	0.75	1.42	-0.12	0.66	0.83	0.44	0.66	5
QN-13	-0.35	0.66	-0.57	0.39	0.64	0.33	0.46	20
QN-14	-0.13	-0.35	0.54	0.44	0.39	0.61	0.47	16
QN-15	0.38	-0.56	0.80	0.57	0.33	0.67	0.52	11
QN-16	-0.57	-0.02	0.62	0.33	0.47	0.63	0.45	21
QN-17	-0.36	-2.41	-0.49	0.38	-0.12	0.35	0.21	36
QN-18	-0.14	-0.65	-0.45	0.44	0.31	0.36	0.38	27
QN-19	-1.61	-0.29	-0.12	0.07	0.40	0.44	0.28	34
QN-20	1.81	0.24	0.19	0.92	0.53	0.52	0.69	3
QN-21	-0.78	0.99	0.36	0.28	0.72	0.56	0.50	12
QN-22	-0.26	0.49	-0.59	0.41	0.59	0.33	0.45	23
QN-23	0.21	-0.50	0.12	0.52	0.35	0.50	0.46	17
QN-24	-0.60	-0.70	-1.11	0.32	0.30	0.20	0.28	33
QN-25	-0.33	-0.80	0.70	0.39	0.28	0.65	0.42	25
QN-26	-0.14	1.11	0.24	0.44	0.75	0.53	0.57	9
QN-27	1.01	1.25	3.88	0.72	0.78	1.44	0.93	1
QN-28	-0.51	-0.49	1.51	0.35	0.35	0.85	0.48	14
QN-29	0.23	0.11	-0.21	0.53	0.50	0.42	0.49	13
QN-30	-1.55	-0.03	0.43	0.09	0.47	0.58	0.34	31
QN-31	-0.26	-0.42	-0.69	0.41	0.37	0.30	0.37	28
QN-32	-1.91	-0.60	0.91	0.00	0.32	0.70	0.29	32
QN-33	-1.32	0.74	-0.11	0.15	0.66	0.44	0.39	26
QN-34	-1.04	1.07	0.03	0.22	0.74	0.48	0.46	18
QN-35	2.01	0.22	-0.38	0.97	0.53	0.38	0.67	4
QN-36	0.06	0.25	-0.39	0.49	0.53	0.38	0.47	15

Table S2. Membership function values and comprehensive ranking of soybean germination stage under 120mM NaCl concentration

Germplasm name	Comprehensive index			Membership function			D-value	Sort
	F1	F2	F3	u1	u2	u3		
QN-1	-0.21	-0.44	-0.21	0.57	0.51	0.57	0.55	22
QN-2	-2.59	0.33	0.99	0.00	0.70	0.86	0.42	34
QN-3	-1.03	1.68	1.33	0.37	1.02	0.94	0.72	11
QN-4	0.61	2.40	-1.17	0.77	1.19	0.34	0.82	4
QN-5	-0.19	0.28	-1.52	0.57	0.69	0.26	0.55	23
QN-6	0.54	-1.13	-0.82	0.75	0.35	0.42	0.54	24
QN-7	0.16	-0.10	-0.73	0.66	0.59	0.45	0.59	19
QN-8	1.54	0.07	0.28	0.99	0.64	0.69	0.80	6
QN-9	1.03	-0.15	2.10	0.87	0.58	1.12	0.82	3
QN-10	0.21	0.85	-0.08	0.67	0.82	0.60	0.71	12
QN-11	-1.51	1.33	-1.36	0.26	0.94	0.29	0.50	29
QN-12	1.59	-0.56	-0.47	1.00	0.48	0.51	0.72	10
QN-13	0.80	-0.68	-0.28	0.81	0.46	0.55	0.63	18
QN-14	1.05	-0.72	0.20	0.87	0.45	0.67	0.68	15
QN-15	0.47	0.79	1.97	0.73	0.81	1.09	0.83	1
QN-16	0.15	-0.19	0.51	0.66	0.57	0.74	0.65	17
QN-17	-2.41	-0.09	-0.03	0.04	0.60	0.61	0.35	36
QN-18	-1.72	0.14	-0.36	0.21	0.65	0.53	0.43	33
QN-19	-0.28	-0.80	-0.78	0.55	0.43	0.43	0.49	30
QN-20	0.64	1.11	-1.54	0.77	0.88	0.25	0.70	13
QN-21	0.97	0.43	0.61	0.85	0.72	0.76	0.79	8
QN-22	0.31	-0.66	-0.29	0.69	0.46	0.55	0.58	20
QN-23	-0.12	1.45	1.54	0.59	0.97	0.99	0.80	7
QN-24	-0.32	-0.53	-0.48	0.54	0.49	0.50	0.52	26
QN-25	-0.49	-0.63	0.43	0.50	0.47	0.72	0.54	25
QN-26	0.41	0.28	0.75	0.72	0.69	0.80	0.72	9
QN-27	0.28	-1.27	0.34	0.69	0.32	0.70	0.56	21
QN-28	-1.10	-0.25	-0.60	0.36	0.56	0.48	0.45	31
QN-29	-0.39	-0.39	-0.87	0.53	0.53	0.41	0.50	28
QN-30	0.79	-1.07	1.02	0.81	0.36	0.86	0.67	16
QN-31	0.82	0.98	-1.93	0.81	0.85	0.16	0.69	14
QN-32	-1.05	-2.13	1.02	0.37	0.11	0.86	0.38	35
QN-33	0.00	-1.17	-0.35	0.62	0.34	0.53	0.51	27
QN-34	-0.30	-1.47	-0.56	0.55	0.27	0.49	0.44	32
QN-35	0.16	1.49	1.44	0.66	0.98	0.96	0.83	2
QN-36	1.17	0.83	-0.10	0.90	0.82	0.60	0.81	5

Table S3. Membership function values and comprehensive ranking of soybean germplasm under 180mM NaCl concentration

Germplasm name	Comprehensive index			Membership function			D-value	Sort
	F1	F2	F3	u1	u2	u3		
QN-1	-0.16	0.22	0.64	0.52	0.61	0.72	0.59	13
QN-2	-1.24	1.42	-2.63	0.25	0.92	-0.11	0.35	34
QN-3	-1.95	1.02	1.11	0.07	0.82	0.84	0.45	30
QN-4	-0.08	0.88	0.70	0.54	0.78	0.74	0.65	8
QN-5	-0.55	0.32	-1.06	0.42	0.64	0.29	0.45	29
QN-6	0.52	-0.48	-0.52	0.69	0.44	0.43	0.56	15
QN-7	0.64	-0.25	-0.20	0.72	0.50	0.51	0.61	12
QN-8	-1.20	-1.55	-0.35	0.26	0.17	0.47	0.28	35
QN-9	-0.51	0.42	-0.10	0.43	0.66	0.53	0.52	22
QN-10	0.56	-1.61	0.54	0.70	0.15	0.69	0.55	18
QN-11	-0.51	1.61	-2.48	0.43	0.96	-0.07	0.46	28
QN-12	1.33	-0.16	-0.88	0.89	0.52	0.34	0.66	7
QN-13	0.32	-0.72	-0.52	0.64	0.38	0.43	0.52	21
QN-14	0.50	-1.14	0.27	0.69	0.27	0.63	0.56	16
QN-15	0.15	-0.65	0.54	0.60	0.40	0.69	0.56	14
QN-16	1.75	0.94	0.93	1.00	0.80	0.79	0.90	1
QN-17	-1.33	0.92	1.20	0.22	0.79	0.86	0.52	20
QN-18	-0.41	-0.17	0.33	0.46	0.52	0.64	0.51	23
QN-19	-1.62	-0.98	-0.97	0.15	0.31	0.31	0.23	36
QN-20	0.71	0.90	-0.71	0.74	0.78	0.38	0.67	6
QN-21	0.88	0.06	0.32	0.78	0.57	0.64	0.69	5
QN-22	-0.44	-0.79	0.65	0.45	0.36	0.72	0.49	26
QN-23	-0.79	0.85	0.68	0.36	0.77	0.73	0.56	17
QN-24	-0.14	-0.47	0.08	0.52	0.44	0.58	0.51	24
QN-25	1.54	-0.55	-1.34	0.95	0.42	0.22	0.64	10
QN-26	0.11	-0.05	-0.49	0.59	0.55	0.44	0.54	19
QN-27	1.59	0.67	0.71	0.96	0.73	0.74	0.85	4
QN-28	0.21	0.59	0.42	0.61	0.71	0.66	0.65	9
QN-29	-2.22	0.60	1.62	0.00	0.71	0.97	0.41	31
QN-30	0.79	-0.59	0.15	0.76	0.41	0.60	0.63	11
QN-31	0.09	-0.11	-1.36	0.58	0.53	0.22	0.48	27
QN-32	-0.07	-2.87	0.44	0.54	-0.16	0.67	0.38	33
QN-33	-0.13	-0.77	0.30	0.53	0.36	0.63	0.51	25
QN-34	-0.79	-0.53	-0.26	0.36	0.42	0.49	0.41	32
QN-35	0.77	1.27	2.02	0.75	0.88	1.07	0.86	3
QN-36	1.65	1.74	0.21	0.98	1.00	0.61	0.90	2

Table S4. Coefficient table of quadratic regression equation in one variable

Germplasm name	RGE			RGR			RGI			RSGC			RTFW			RVI			RRL		
	a	b	c	a	b	c	a	b	c	a	b	c	a	b	c	a	b	c	a	b	c
QN-1	-0.056	0.025	0.982	-0.049	0.016	1.004	-0.022	-0.143	0.993	-0.010	-0.201	0.990	0.016	-0.197	1.005	0.109	-0.631	0.992	0.086	-0.526	0.998
QN-2	0.098	-0.587	0.978	-0.008	-0.261	0.991	-0.067	-0.071	1.013	0.036	-0.362	0.991	-0.066	-0.033	0.994	0.044	-0.466	1.029	0.043	-0.395	1.028
QN-3	-0.134	0.125	1.002	-0.178	0.253	0.997	-0.082	-0.034	1.004	-0.140	0.146	1.000	-0.028	-0.028	0.998	-0.011	-0.311	1.031	-0.040	-0.166	1.024
QN-4	-0.129	0.208	0.976	-0.067	0.084	0.978	-0.062	-0.023	0.976	-0.098	0.130	0.976	0.035	-0.259	1.011	-0.092	-0.053	1.033	-0.140	0.139	1.052
QN-5	-0.145	0.202	1.020	-0.110	0.143	0.996	-0.053	-0.092	1.012	-0.116	0.119	1.011	0.023	-0.265	1.012	-0.013	-0.308	1.045	-0.048	-0.157	1.039
QN-6	-0.098	0.115	0.991	-0.006	-0.049	0.981	-0.041	-0.085	1.012	-0.067	0.047	0.989	0.045	-0.309	1.008	0.063	-0.514	1.018	0.045	-0.430	1.014
QN-7	-0.041	0.020	0.991	-0.015	-0.024	1.001	-0.031	-0.092	1.002	-0.030	-0.122	1.000	0.020	-0.231	1.007	0.049	-0.467	1.012	0.022	-0.359	1.012
QN-8	-0.247	0.488	0.968	-0.187	0.381	0.986	-0.162	0.227	0.986	-0.228	0.438	0.978	-0.002	-0.176	0.989	0.044	-0.448	0.983	0.048	-0.432	0.977
QN-9	-0.120	0.183	0.951	-0.104	0.154	0.955	-0.113	0.112	0.978	0.130	-0.662	1.099	0.017	-0.212	0.983	0.011	-0.354	1.009	0.011	-0.313	1.015
QN-10	-0.048	0.027	0.965	-0.015	0.027	0.997	0.004	-0.210	0.973	0.023	-0.209	0.973	0.036	-0.257	0.983	0.054	-0.471	0.986	-0.029	-0.210	1.002
QN-11	-0.037	-0.130	1.000	-0.034	-0.121	1.010	-0.049	-0.100	1.019	-0.040	-0.131	1.020	-0.069	-0.035	1.039	-0.053	-0.197	1.065	-0.084	-0.043	1.054
QN-12	-0.112	0.250	0.997	-0.077	0.174	0.994	-0.095	0.131	0.997	-0.079	0.064	0.996	-0.014	-0.149	1.010	-0.023	-0.270	1.053	-0.007	-0.290	1.048
QN-13	-0.126	0.187	0.989	-0.042	0.086	0.993	-0.055	-0.047	0.992	-0.082	0.076	0.986	0.011	-0.214	1.003	0.071	-0.531	1.003	0.049	-0.438	1.007
QN-14	-0.071	0.104	0.971	-0.078	0.132	0.986	-0.052	-0.003	0.978	-0.036	-0.067	0.978	-0.033	-0.077	1.027	0.086	-0.569	0.990	0.068	-0.501	0.997
QN-15	-0.099	0.120	0.973	-0.014	-0.041	0.980	-0.079	0.046	0.977	-0.078	0.071	0.973	-0.046	-0.024	1.003	0.018	-0.366	0.998	0.001	-0.287	1.005
QN-16	-0.013	-0.015	1.007	0.003	-0.053	0.998	-0.001	-0.128	1.007	0.030	-0.242	1.001	0.038	-0.250	1.004	0.053	-0.457	1.016	0.030	-0.361	1.016
QN-17	0.038	-0.330	0.986	0.031	-0.362	1.039	0.035	-0.347	0.980	0.049	-0.396	0.994	0.056	-0.295	1.008	0.113	-0.652	0.994	0.039	-0.395	1.018
QN-18	0.000	-0.204	1.013	0.013	-0.236	1.016	-0.001	-0.230	1.016	-0.009	-0.197	1.020	0.043	-0.276	0.999	0.080	-0.562	1.010	0.033	-0.388	1.005
QN-19	-0.126	0.129	0.983	-0.147	0.194	0.976	-0.086	-0.019	0.992	-0.028	-0.193	0.986	0.035	-0.295	0.993	0.099	-0.616	0.986	0.074	-0.512	0.983
QN-20	-0.100	0.143	0.989	-0.005	0.008	0.999	-0.047	-0.073	0.991	-0.078	0.091	0.992	-0.050	-0.026	1.037	-0.019	-0.286	1.048	-0.056	-0.139	1.061
QN-21	-0.075	0.097	0.995	-0.105	0.223	0.985	-0.088	0.115	0.994	-0.064	0.010	0.992	0.007	-0.178	0.991	0.030	-0.390	1.003	0.042	-0.395	1.001
QN-22	-0.107	0.126	0.991	-0.093	0.140	0.988	-0.067	-0.043	1.003	-0.075	0.033	0.989	0.037	-0.282	1.007	0.057	-0.495	1.009	0.040	-0.401	1.006
QN-23	-0.070	-0.014	0.972	-0.110	0.156	0.977	-0.090	0.058	0.974	-0.091	0.033	0.985	-0.033	-0.062	0.990	0.024	-0.372	0.992	0.024	-0.317	1.003
QN-24	-0.051	-0.042	0.987	-0.043	0.050	0.994	-0.005	-0.201	0.992	0.014	-0.249	0.988	0.033	-0.268	0.997	0.107	-0.630	0.992	0.067	-0.481	0.999
QN-25	0.021	-0.213	1.013	0.072	-0.251	0.998	-0.008	-0.137	1.011	0.031	-0.218	1.011	-0.007	-0.181	1.008	0.093	-0.584	0.997	0.069	-0.490	0.993
QN-26	-0.118	0.150	0.989	-0.039	0.016	1.025	-0.110	0.122	0.992	-0.100	0.112	0.991	-0.010	-0.141	1.011	-0.013	-0.292	1.027	-0.008	-0.271	1.024

QN-27	-0.095	0.191	1.040	0.001	-0.206	0.979	-0.101	0.155	1.072	-0.054	0.039	1.038	-0.026	-0.076	1.047	-0.018	-0.296	1.083	0.046	-0.442	1.039
QN-28	-0.079	0.033	1.019	0.011	-0.177	1.029	0.022	-0.249	1.013	0.019	-0.285	1.002	-0.014	-0.143	1.038	0.100	-0.601	1.005	0.064	-0.458	1.006
QN-29	-0.191	0.243	0.993	-0.087	0.054	0.993	-0.100	0.021	0.996	-0.086	-0.042	0.989	0.060	-0.323	1.024	0.027	-0.421	1.024	0.023	-0.351	1.026
QN-30	-0.062	0.076	0.979	-0.067	0.094	0.985	-0.046	-0.028	0.998	-0.014	-0.097	0.975	0.013	-0.187	0.986	0.122	-0.669	0.986	0.115	-0.628	0.986
QN-31	-0.095	0.160	0.975	-0.108	0.221	0.952	-0.050	-0.043	0.979	-0.024	-0.149	0.992	-0.003	-0.166	1.000	0.076	-0.534	0.979	0.047	-0.425	0.983
QN-32	-0.006	-0.151	0.981	0.035	-0.231	1.034	0.001	-0.221	1.011	-0.006	-0.221	1.010	0.031	-0.279	0.999	0.115	-0.667	1.001	0.081	-0.542	1.003
QN-33	-0.074	0.028	1.007	-0.055	0.053	1.001	-0.066	-0.016	1.005	-0.060	-0.060	1.010	0.065	-0.364	1.000	0.079	-0.550	1.000	0.071	-0.491	0.996
QN-34	-0.070	0.000	1.000	-0.094	0.099	0.994	-0.088	0.005	1.021	-0.106	0.036	1.039	0.049	-0.331	0.999	0.081	-0.570	1.014	0.087	-0.543	1.005
QN-35	-0.044	0.058	0.996	-0.028	0.043	0.988	-0.031	-0.095	0.991	-0.076	0.042	0.998	-0.021	-0.049	1.007	-0.058	-0.156	1.053	-0.101	0.021	1.068
QN-36	-0.071	0.150	0.980	-0.030	0.051	0.982	-0.044	0.010	0.983	-0.043	-0.029	0.992	0.013	-0.187	1.004	0.045	-0.412	0.992	0.041	-0.370	0.999

Note: The function parameter values (a, b, c) are based on the least squares method and are used to fit a quadratic function (Y=ax2+bx+c) to the RGR, RGE, RGI, RVI, RSGC, RTFW, and RRL parameters of each strain at different salt concentrations.

Table S5. Membership function values and comprehensive ranking of soybean germplasm under semi lethal concentration (LC50)

Germplasm name	Comprehensive index			Membership function			D-value	Sort
	F1	F2	F3	u1	u2	u3		
QN-1	-0.27	-0.87	0.77	0.52	0.37	0.78	0.52	26
QN-2	-2.34	0.54	-0.99	0.00	0.72	0.34	0.28	36
QN-3	-1.64	0.49	2.54	0.17	0.71	1.22	0.53	24
QN-4	0.21	2.95	-0.70	0.64	1.33	0.41	0.81	3
QN-5	-0.35	0.91	-1.29	0.50	0.81	0.26	0.55	20
QN-6	0.64	-0.46	-0.91	0.74	0.47	0.36	0.59	18
QN-7	0.56	-0.26	-0.34	0.73	0.52	0.50	0.62	13
QN-8	0.18	-0.56	-0.58	0.63	0.45	0.44	0.54	22
QN-9	-0.61	0.35	-0.08	0.43	0.67	0.56	0.53	23
QN-10	0.59	0.16	-0.71	0.73	0.62	0.41	0.64	11
QN-11	-1.17	1.78	-1.23	0.29	1.03	0.28	0.52	27
QN-12	1.65	0.31	-0.57	1.00	0.66	0.44	0.80	5
QN-13	0.73	-0.51	-0.96	0.77	0.46	0.35	0.60	17
QN-14	0.79	-1.37	0.92	0.78	0.24	0.82	0.62	12
QN-15	0.40	0.28	0.79	0.68	0.66	0.78	0.69	9
QN-16	1.15	0.33	0.58	0.87	0.67	0.73	0.79	6
QN-17	-2.21	-0.60	1.43	0.03	0.43	0.94	0.32	34
QN-18	-1.01	-0.60	0.56	0.33	0.44	0.72	0.43	30
QN-19	-1.22	-1.24	-0.80	0.28	0.27	0.38	0.30	35
QN-20	0.77	0.99	0.11	0.78	0.83	0.61	0.77	7
QN-21	1.10	0.28	0.13	0.86	0.65	0.62	0.75	8
QN-22	0.00	-0.22	-0.61	0.59	0.53	0.43	0.54	21
QN-23	-0.61	1.08	0.47	0.43	0.86	0.70	0.61	14
QN-24	-0.19	-0.69	-0.76	0.54	0.41	0.39	0.47	29
QN-25	0.84	-0.59	-1.07	0.80	0.44	0.32	0.60	16
QN-26	0.45	0.52	-0.09	0.70	0.71	0.56	0.68	10
QN-27	1.15	-0.61	2.62	0.87	0.43	1.24	0.80	4
QN-28	-0.48	-0.26	0.34	0.47	0.52	0.67	0.52	25
QN-29	-1.60	0.09	0.41	0.18	0.61	0.69	0.40	32
QN-30	0.92	-1.66	0.75	0.82	0.17	0.77	0.61	15
QN-31	0.35	-0.73	-0.05	0.67	0.40	0.57	0.57	19
QN-32	-0.41	-1.45	-0.75	0.48	0.22	0.40	0.39	33
QN-33	0.09	-0.58	-0.96	0.61	0.44	0.35	0.51	28
QN-34	-0.48	-0.82	-1.01	0.46	0.38	0.33	0.42	31
QN-35	0.58	2.13	1.69	0.73	1.12	1.01	0.90	1
QN-36	1.46	0.91	0.39	0.95	0.81	0.68	0.86	2

Table S6. 36 soybean germplasm names

Code	Germplasm name	Breeding Institution
QN-1	QiHuang34	Crop Research Institute of Shandong Academy of Agricultural Sciences
QN-2	QingNong641	Qingdao Agricultural University
QN-3	FenDou93	Economic Crop Research Institute of Shanxi Academy of Agricultural Sciences
QN-4	QingNong188	Qingdao Agricultural University
QN-5	QingNong187	Qingdao Agricultural University
QN-6	184	Qingdao Agricultural University
QN-7	181	Qingdao Agricultural University
QN-8	180	Qingdao Agricultural University
QN-9	173	Qingdao Agricultural University
QN-10	171	Qingdao Agricultural University
QN-11	170	Qingdao Agricultural University
QN-12	167	Qingdao Agricultural University
QN-13	166	Qingdao Agricultural University
QN-14	160	Qingdao Agricultural University
QN-15	158	Qingdao Agricultural University
QN-16	153	Qingdao Agricultural University
QN-17	151	Qingdao Agricultural University
QN-18	149	Qingdao Agricultural University
QN-19	148	Qingdao Agricultural University
QN-20	144	Qingdao Agricultural University
QN-21	143	Qingdao Agricultural University
QN-22	141-1	Qingdao Agricultural University
QN-23	141-2	Qingdao Agricultural University
QN-24	140	Qingdao Agricultural University
QN-25	137	Qingdao Agricultural University
QN-26	134	Qingdao Agricultural University
QN-27	130	Qingdao Agricultural University
QN-28	120	Qingdao Agricultural University
QN-29	105	Qingdao Agricultural University
QN-30	80	Qingdao Agricultural University
QN-31	62	Qingdao Agricultural University
QN-32	QN-23-31	Qingdao Agricultural University
QN-33	SyF3-15	Qingdao Agricultural University
QN-34	KeDou35	Institute of Genetics and Developmental Biology, Chinese Academy of Sciences
QN-35	QingNong141	Qingdao Agricultural University
QN-36	ShengDou5	Shandong Shengfeng Seed Industry Technology Co., Ltd
